# Spatio-temporal analysis on enterovirus cases through integrated surveillance in Taiwan

**DOI:** 10.1186/1471-2458-14-11

**Published:** 2014-01-08

**Authors:** Ta-Chien Chan, Jing-Shiang Hwang, Rung-Hung Chen, Chwan-Chuen King, Po-Huang Chiang

**Affiliations:** 1 Research Center for Humanities and Social Sciences, Academia Sinica, Taipei 115, Taiwan; 2 Institute of Statistical Science, Academia Sinica, Taipei 115, Taiwan; 3 Department of Medicine, College of Medicine, National Taiwan University (NTU), Taipei 100, Taiwan; 4 Institute of Epidemiology and Preventive Medicine, College of Public Health, NTU, Taipei 100, Taiwan; 5 Institute of Population Health Sciences, National Health Research Institutes, Zhunan 350, Taiwan

**Keywords:** Enterovirus, Epidemiologic characteristics, Surveillance, Taiwan

## Abstract

**Background:**

Severe epidemics of enterovirus have occurred frequently in Malaysia, Singapore, Taiwan, Cambodia, and China, involving cases of pulmonary edema, hemorrhage and encephalitis, and an effective vaccine has not been available. The specific aim of this study was to understand the epidemiological characteristics of mild and severe enterovirus cases through integrated surveillance data.

**Methods:**

All enterovirus cases in Taiwan over almost ten years from three main databases, including national notifiable diseases surveillance, sentinel physician surveillance and laboratory surveillance programs from July 1, 1999 to December 31, 2008 were analyzed. The Pearson’s correlation coefficient was applied for measuring the consistency of the trends in the cases between different surveillance systems. Cross correlation analysis in a time series model was applied for examining the capability to predict severe enterovirus infections. Poisson temporal, spatial and space-time scan statistics were used for identifying the most likely clusters of severe enterovirus outbreaks. The directional distribution method with two standard deviations of ellipse was applied to measure the size and the movement of the epidemic.

**Results:**

The secular trend showed that the number of severe EV cases peaked in 2008, and the number of mild EV cases was significantly correlated with that of severe ones occurring in the same week [r = 0.553, p < 0.01]. These severe EV cases showed significantly higher association with the weekly positive isolation rates of EV-71 than the mild cases [severe: 0.498, p < 0.01 vs. mild: 0.278, p < 0.01]. In a time series model, the increase of mild EV cases was the significant predictor for the occurrence of severe EV cases. The directional distribution showed that both the mild and severe EV cases spread extensively during the peak. Before the detected spatio-temporal clusters in June 2008, the mild cases had begun to rise since May 2008, and the outbreak spread from south to north.

**Conclusions:**

Local public health professionals can monitor the temporal and spatial trends plus spatio-temporal clusters and isolation rate of EV-71 in mild and severe EV cases in a community when virus transmission is high, to provide early warning signals and to prevent subsequent severe epidemics.

## Background

In July 2012, 54 children died of infections with enterovirus-71 (EV-71) in Cambodia [1]. Before the laboratory results came back, the media called it a mystery disease, which made numerous Asian parents worried. In fact, severe epidemics of enterovirus have occurred frequently in Asia, including Malaysia [2], Singapore [3], Taiwan [4,5] and China [6]. The clinical severity varied from asymptomatic to mild symptoms [hand-foot-mouth disease (HFMD) and herpangina], severe pulmonary edema, hemorrhage and encephalitis [7]. The EV-71 infections involved 6-29% asymptomatic infection, 13-32% non-specific viral syndrome, and 37%-40% HFMD/ herpangina symptoms [8]. Among the non-polio enterovirus serotypes, EV-71, which has caused severe clinical illness and many fatal cases [9], and particularly a high risk of poor prognosis for children under one year of age [7], has become one of the most important public health concerns since the World Health Organization (WHO) launched the “global polio eradication initiative” program in 1988 [10]. However, children with an asymptomatic and mild infection of EV-71 still can carry the virus to transmit to others [11]. During 1998 ~ 2005, 37.98% (588/1,584) of severe EV cases in Taiwan had only encephalitis complications, and 22.8% (353/1,584) had encephalitis with pulmonary edema or hemorrhage [7]. In other words, whether increasing numbers of mild EV cases would provide a possible sentinel signal of the early stage of an epidemic is worth investigating, particularly as a vaccine and more effective drugs for EV-71 have not been available [12,13]. Therefore, using an integrated surveillance system to monitor the enterovirus activity and fully understanding the difference in epidemiological characteristics between mild and severe enterovirus cases will be the most important prevention and control measures in public health.

After the first nationwide epidemic of EV-71 occurred in Taiwan in 1998, there were another three cross-county epidemics in 2000–2001, 2004–2005, 2008–2009 [4]. The question is, what are the important epidemiological characteristics that will be helpful in surveillance of EV-71 to minimize the severity of future epidemics? The specific aims of this study were: (1) to elucidate the spatio-temporal correlations between the mild and severe enterovirus cases through integrating the data of the three enterovirus-related surveillance systems, including the sentinel physician, national notifiable diseases and laboratory surveillance systems in Taiwan, (2) to find out the feasibility of establishing an early warning signal using the increasing numbers of mild EV-71 cases and their lag time periods to appearance of severe EV-71 cases, and (3) to evaluate the trends of severe EV-71 cases over a 9.5-year period for providing better recommendations on public health efforts in the future. With full understanding of the epidemiological characteristics, hopefully we can develop better measures and indicators from mild cases to provide early warning signals and thus minimizing subsequent numbers of severe cases.

## Methods

### Surveillance for enteroviruses

Three types of databases from the Taiwan Centers for Diseases Control (Tw-CDC), including national notifiable diseases surveillance, sentinel physician surveillance and laboratory surveillance from July 1, 1999 to December 31, 2008, were used in this study. Mild enterovirus infections including hand-foot-mouth disease or herpangina were reported by the sentinel physicians each week. For enterovirus infections with severe complications, the clinicians are required to report to Tw-CDC through the national notifiable diseases surveillance system within one week. The reporting definition of severe EV cases must meet one of two criteria: (1) patients with typical hand-foot-mouth disease (HFMD) or herpangina, or epidemiologically-linked enterovirus infected patients accompanied by myoclonic jerks or encephalitis, acute flaccid paralysis, acute hepatitis, myocarditis, or cardiopulmonary failure, and (2) infants under 3 months of age with myocarditis, hepatitis, encephalitis, thrombocytopenia, multiple organ dysfunction syndrome (MODS) or sepsis symptoms, excluding the infection of bacteria or other pathogens [14]. For the sentinel-physician surveillance system, involving voluntary-based sentinel physicians in Taiwan, there were around 670 sentinel physicians from 510 clinics and 71 hospitals [15]. The cases with HFMD or herpangina reported through this system were compiled on a weekly basis. For virological surveillance data, all those specimens that were collected by sentinel physicians for highly suspected EV cases and sent to the 12 regional contracted laboratories of the Tw-CDC were used to examine the viral types, including EV-71, coxasackievirus groups A/B, echovirus, and other enterovirus [16].

### Statistical analysis

In this study, the temporal unit was the week, and the spatial unit was the city or county. To compare the trends between the mild and severe EV cases and between the isolation rates of different serotypes of EV and the number of severe EV cases, Pearson’s correlation coefficients were applied for measuring the consistency of the weekly data with the statistical software, SPSS (Version 18.0, SPSS Science, Chicago, IL). Because the data of sentinel physicians were collected on a weekly basis, the counts of the severe EV cases and virological results were also aggregated into a weekly basis for better comparison. Weekly numbers of severe cases and EV-positive isolation rates were used to compare their temporal trends, whereas the weekly incidence rates of severe EV-cases were employed to describe spatial distributions over time. A lag effect between mild and severe EV cases was taken into account to find out whether mild EV cases occurred earlier or later than severe EV cases, in order to determine whether mild cases might serve as early warning signals for severe cases. In addition, we applied cross-correlation analysis in a time series model to see whether there was any conditional correlation among the severe and mild EV cases and four types of EV isolation. An ARMA model (Autoregressive moving average model) was fit using the SAS Release 9.2 software (Cary, NC). The selection of the autoregressive (AR) and moving average (MA) was based on the minimum information criterion (MINIC) method [17].

Because the mild and severe EV cases were from different surveillance systems, the age definition of the EV cases we used was different. The mild EV cases were from sentinel surveillance, which had aggregated reported cases without age information. Thus, the age definition of mild EV was all ages. The severe EV cases were from the notifiable infectious disease system, which had complete age information. Because most of the high risk population of severe EV cases was children during the study period, we selected the cases aged equal or less than 14 years (99.7%, 1,512/1,517, median age = 21.5 months) for further cluster analysis. The cumulative incidence of the pediatric severe EV cases was calculated with the corresponding mid-year population from July 1999 to 2008. Most likely clusters with high incidence of severe EV cases were detected retrospectively using spatial statistic, temporal statistic, and space-time scan statistic implemented in SaTScan v.9.1.1.[18]. The population data throughout the study period in each city or county were collected from Taiwan’s National Statistics website (http://ebas1.ebas.gov.tw/pxweb/Dialog/statfile9.asp). All the 9.5 years cases’ data were used, with a maximum cluster population size of 5% to minimize false clusters, and a maximum temporal window of one month to examine the temporal-cluster using the software of SaTScan. The analyses of data with case counts were carried out using the Poisson probability model (for a few pediatric EV cases among the child population) with 999 Monte Carlo replications to test for the presence of statistically significant spatial clusters [19], and choosing the parameter for no geographic overlapping clusters to avoid repeated counting. After identifying the space-time clusters of the severe EV cases, the corresponding data of the mild EV cases from sentinel physician surveillance were further analyzed for consistent temporally increasing trends or even earlier increasing trends. Space-Time permutation was applied for detecting the mild EV clusters due to the lack information of the population at risk [20]. The directional distribution method was employed, with two standard deviations of the ellipse size weighted by either the mild EV cases per doctor or the number of severe EV cases [21]. Then, we applied GIS software (ArcMap, version9.3; ESRI Inc.,Redlands, CA, USA) for visualization of all detected statistically significant clusters.

## Results

### Age distribution of mild and severe EV cases

**Figure 1:**
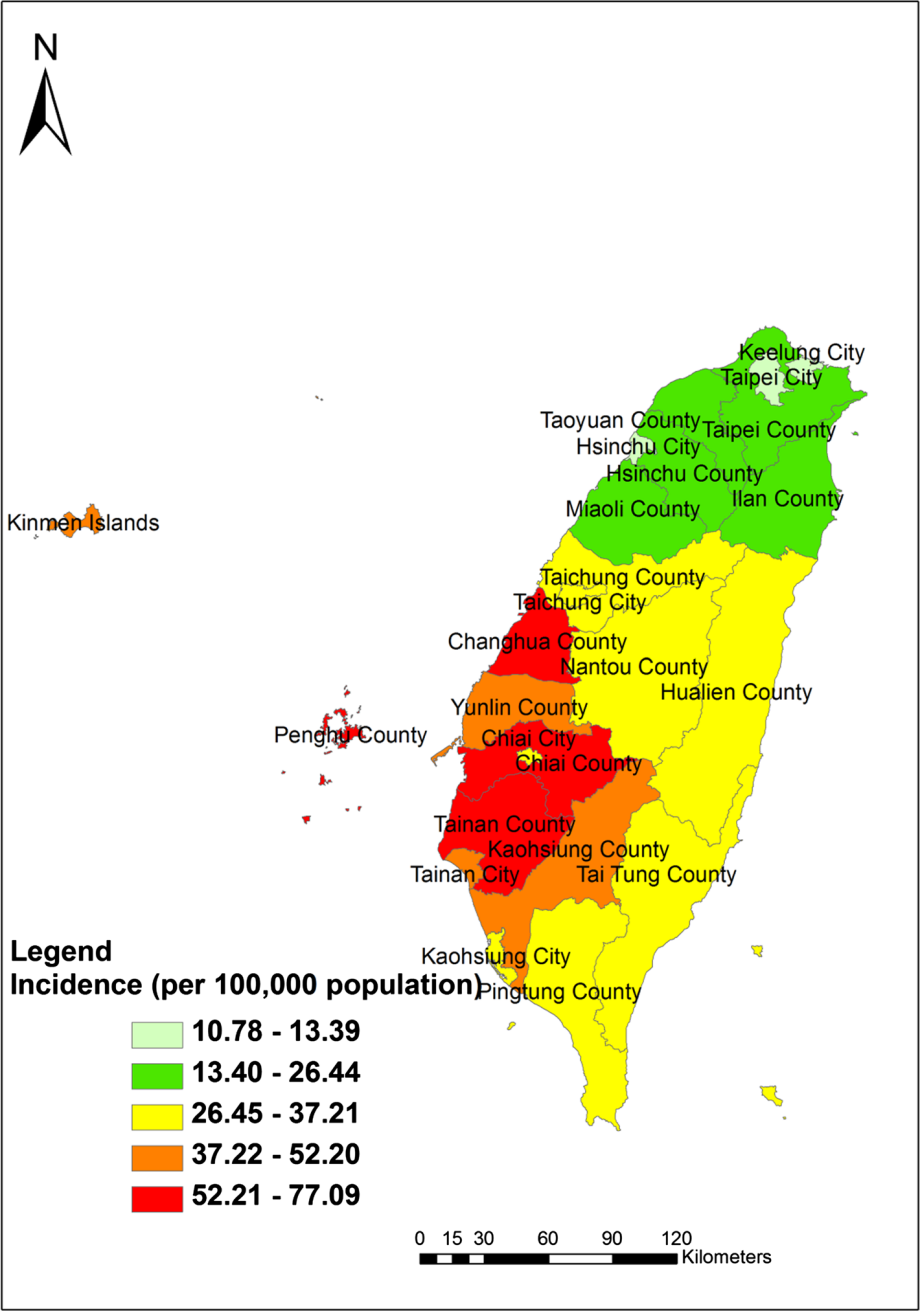
Geographical distribution in the cumulative incidence of pediatric severe EV cases (aged 0–14) in Taiwan from July 1999 to December 2008.

Among the 1,517 severe EV cases notified to the Taiwan-CDC during the study period, the mean age was 27 months [mean ± standard deviation (S.D.) = 27 ± 25.3], 61.4% were male, 71% were hospitalizations or referrals, and 12% were fatal. In the virological surveillance database, the mean age of EV-71 cases was 42.51 months (S.D. = 41.8 months, n = 2,358) and that of Non-EV-71 cases was 87.7 months (S.D. = 145.4 months, n = 47,645). Most of the severe EV cases were aged less than 14 years, [99.7% (1,512/1,517)] which was the group used for cluster analysis. The distribution of incidence of severe EV aged 0–14 during the study period was high in central and southern Taiwan and surrounding islands including Penghu County and Kinmen County (Figure 1).

### Temporal relationship between mild and severe EV cases

**Figure 2:**
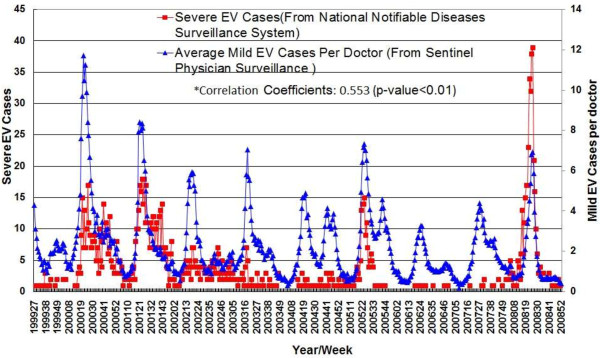
**The temporal trend between severe EV cases and average mild EV cases per doctor.** The X axis is the time in 6 digits; the first four digits are the year, and the last 2 digits are the week number.

Temporal analysis in Figure 2 found that the mild EV cases and all the severe EV cases occurring in the same week were significantly correlated (Pearson’s correlation coefficient = 0.553, p < 0.01). Severe EV cases had two peaks starting from 2000, but the second peak was less pronounced beginning in 2003. After considering the lag effect, the correlation between mild EV cases 1 and 2 weeks ahead and the later severe EV cases was 0.523 (p < 0.01) and 0.467 (p < 0.01), respectively. On the other hand, the correlation between severe cases which were 1 or 2 weeks earlier and the subsequent mild EV cases was 0.554 (p < 0.01) and 0.512 (p < 0.01) in Table 1, respectively. The highest correlation coefficients shifted from the mild EV cases earlier to severe EV cases earlier since 2002–2003.

**Table 1:** Temporal correlation between mild EV and severe EV cases in Taiwan by different years, from July 1, 1999 to December 31, 2008

	**July 1 ~ Dec. 31, 1999**	**2000**	**2001**	**2002**	**2003**	**2004**	**2005**	**2006**	**2007**	**2008**	**Overall**
S-1week	-0.174	.584^**^	.747^**^	.429^**^	.488^**^	.544^**^	.783^**^	.596^**^	0.194	.975**	.554^**^
S-2week	-0.093	.510^**^	.664^**^	.331^*^	.418^**^	.505^**^	.753^**^	.565^**^	0.147	.919^**^	.512^**^
S-M	-0.078	.609^**^	.792^**^	.431^**^	.437^**^	.459^**^	.754^**^	.595^**^	0.187	.949^**^	.553^**^
M-1week	-0.177	.648^**^	.806^**^	.387^**^	0.243	.418^**^	.660^**^	.542^**^	0.203	.849^**^	.523^**^
M-2week	-0.238	.640^**^	.763^**^	.341^*^	0.09	.390^**^	.528^**^	.482^**^	0.269	.704^**^	.467^**^
EV-71%	10.89	32.66	32.16	15.78	5.61	33.78	18.86	0.16	1.00	32.92	5.81
CA%	35.64	34.68	29.12	36.70	63.18	10.92	41.21	70.87	88.24	51.02	11.75
CB%	43.56	16.78	4.73	23.17	9.23	51.26	35.79	12.46	2.94	14.64	4.88
ECHO%	15.84	16.11	34.07	24.89	22.07	4.03	4.14	16.67	7.88	1.45	5.17

S-1 week: Severe EV Earlier than Mild EV Cases with lag 1 week.

S-2 week: Severe EV Earlier than Mild EV Cases with lag 2 weeks.

S-M: Mild and Severe EV Cases in the same week.

M-1 week: Mild EV Earlier than Severe EV Cases with lag 1 week.

M-2 week: Mild EV Earlier than Severe EV Cases with lag 2 weeks.

CA: Coxsackievirus A Virus; CB: Coxsackievirus B Virus; ECHO: Echovirus.

*p-value < 0.05.

**p-value < 0.01.

**Figure 3:**
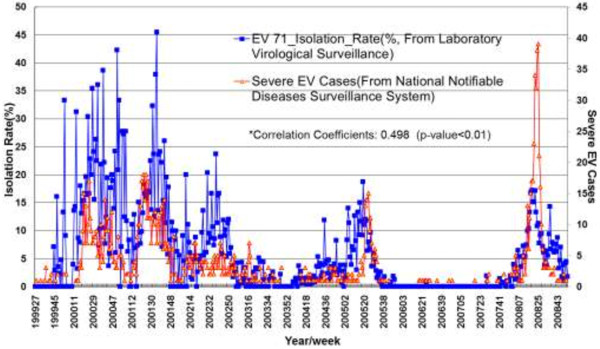
The temporal trend between EV-71 isolation rate and severe EV cases.

In Table 2, the correlation among mild and severe EV cases and the isolation rates of the major four types of non-polio enterovirus are shown. The isolation rates of EV-71 were highly correlated with the occurrence of severe EV cases (r = 0.498, p < 0.01). Their temporal pattern is shown in Figure 3. Such weekly correlation coefficients were much lower for other types of EV [0.184 (p < 0.01) for coxsackie A virus, 0.124 (p < 0.01) for coxsackie B virus and 0.206 (p < 0.01) for ECHO virus].

**Table 2:** The correlation among mild, severe EV and major four types of EV isolation rate

	**Severe EV Cases**	**Mild EV Cases**	**EV-71 Isolation Rate**	**CAV Isolation Rate**	**CBV Isolation Rate**	**ECHO Isolation Rate**
Severe EV Cases	1	.553**	.498**	0.184**	0.124**	0.206**
Mild EV Cases	-	1	.278**	0.18**	0.11*	0.13**
EV-71 Isolation Rate	-	-	1	0.19**	0.16**	0.29**
CAV Isolation Rate	-	-	-	1	0.14**	.064
CBV Isolation Rate	-	-	-	-	1	-.041
ECHO Isolation Rate	-	-	-	-	-	1

CA: Coxsackievirus A Virus; CB: Coxsackievirus B Virus; ECHO: Echovirus.

**p-value < 0.01, *p-value < 0.05.

In Table 3, the dependent variable was the severe EV cases each week. The explanatory variables were the mild EV cases, the isolation rates of EV-71, Coxsackievirus A Virus, Coxsackievirus B Virus, and Echovirus in each week. In the first model, without considering the ARMA effect, only mild EV cases (coefficient = 1.23, p < 0.0001) and the EV-71 isolation rate (coefficient = 0.22, p < 0.0001) were significant predictors. In the second model, considering ARMA (4,0) which was selected by MINIC function in SAS, only the AR effects and mild EV (coefficient = 1.23, p < 0.0001) were significant predictors. On the other hand, we also switched mild EV cases as the dependent variable and severe EV cases as an explanatory variable. After considering the AR effect, severe EV cases could not be significant predictors for mild EV cases (p = 0.86, data are not shown).

**Table 3:** The cross-correlation analysis among severe EV cases, mild EV cases and four types of EV isolation rate

**Variable**	**Estimate**	**Standard Error**	**p-value**
**Model 1**			
Intercept	-1.40	0.33	<.0001***
Mild EV cases	1.23	0.10	<.0001***
EV-71	0.22	0.02	<.0001***
CAV	0.02	0.02	0.37
CBV	0.01	0.02	0.64
ECHO	0.03	0.02	0.19
**Model 2**			
Intercept	-0.42	0.97	0.67
AR1,1	0.56	0.05	<.0001***
AR1,2	0.27	0.05	<.0001***
AR1,3	0.17	0.05	0.0009**
AR1,4	-0.11	0.05	0.0134*
Mild EV cases	1.23	0.16	<.0001***
EV-71	0.00	0.02	0.80
CAV	0.00	0.01	0.85
CBV	0.00	0.02	0.96
ECHO	-0.01	0.02	0.55

CA: Coxsackievirus A Virus; CB: Coxsackievirus B Virus; ECHO: Echovirus.

***p-value < 0.0001, **p-value < 0.01, *p-value < 0.05.

### Spatial relationship between mild and severe EV Cases

To fully understand the temporal, spatial and tempo-spatial distributions of EV cases, we then monitored the trends in EV cases using these three methods separately. With the temporal scan method alone, the temporal cluster was only detected in June 2008, which had the highest number of monthly severe EV cases (n = 137) during the study period. In the further analysis, the number of the severe EV cases from April 2008 to June 2008 was 277, which was also the highest value for any three-month period during the study period.

**Figure 4:**
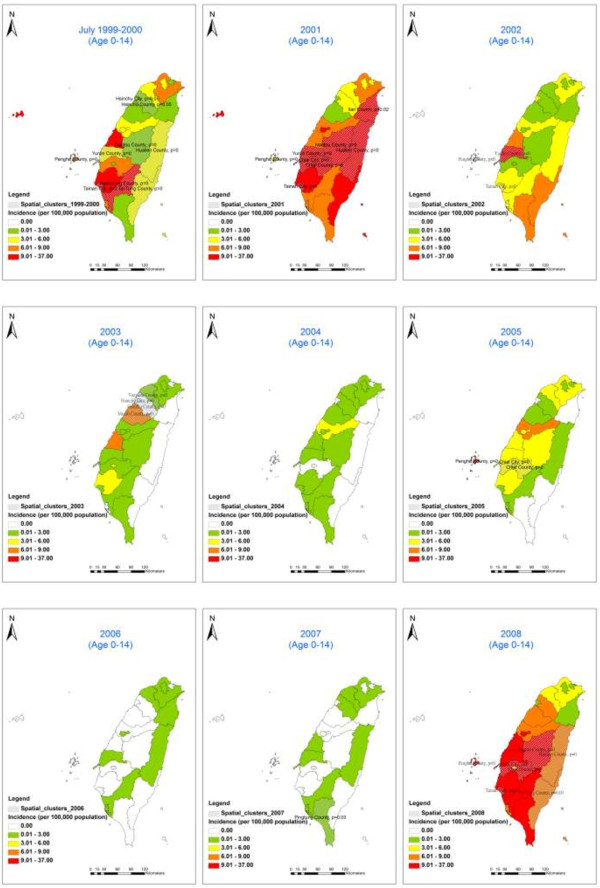
Spatial clusters of severe EV cases (aged 0–14) in Taiwan from July 1999 to December 2008.

**Figure 5:**
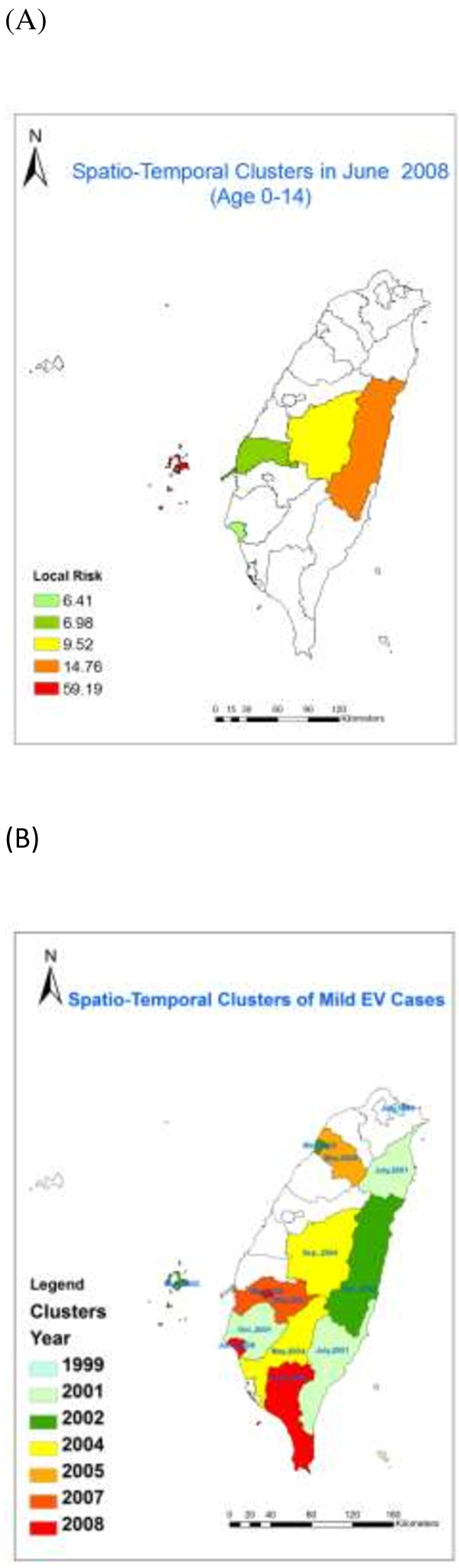
**Spatio-temporal clusters of mild and severe EV cases from July 1999 to Deccember 2008. (A)** Severe EV cases aged from 0 to 14; **(B)** Mild EV cases from all ages.

**Figure 6:**
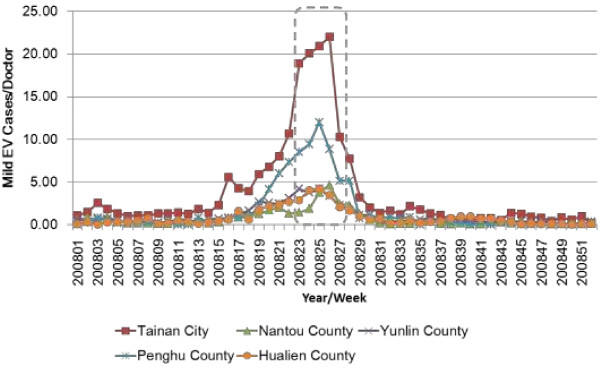
Temporal trend of mild EV cases during spatio-temporal clusters of severe EV cases in 2008: Vertical gray dashed lines represent the time period in June 2008.

**Figure 7:**
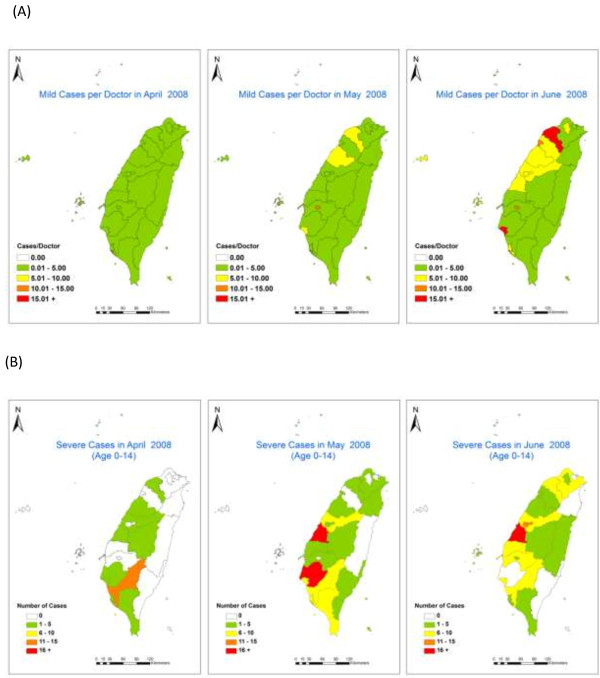
Spatial distribution of mild (A) and severe (B) enterovirus cases from the beginning to the peaking month (April 2008-June 2008).

With the spatial scan method alone, there were two years without statistical significant spatial clusters (i.e. 2004, and 2006), while the other seven years had statistical significant spatial clusters: July 1999–2000, 2001, 2002, 2003, 2005, 2007 and 2008. Throughout the study period (Figure 4), Penghu County in July 1999–2000, and 2008 had the highest local spatial risk (25.2 and 22.8, p < 0.05). With the integrated space-time scan method, the five cities or counties which had the highest number (137) of severe cases throughout the study period and the highest number (6) of fatal cases in 2008 were identified (Figure 5A). Three space-time mild EV clusters were detected in 2008 (Figure 5B). In Tainan City, consistent mild and severe EV clusters were both detected in June 2008. Another two mild EV clusters in 2008 were found earlier, in April and May. Penghu County also had the highest local tempo-spatial risk among these (59.19, p < 0.01). Then, we analyzed the temporal trend in mild EV cases per doctor in 2008. In Figure 6, the period of June 2008, which is marked as the gray dashed square, was also the peak of the mild EV cases. However, the increasing trend of severe EV cases surged starting in week 15, 2008 (April 4, 2008) and mild EV cases also surged in the following week (April 11, 2008). In Figure 7(A), mild EV cases began to flare up in May 2008 in northern and southern Taiwan and peaked in June 2008 in northern and southern Taiwan. In Figure 7(B), severe EV cases began to flare up in April 2008 in southern Taiwan and moved upward to central Taiwan in June 2008. The geographical size and the direction of movement of the EV epidemic from April 2008 to June 2008 was getting larger and moving from the south to the north (Table 4).

**Table 4:** The size and direction of movement during the EV epidemic in 2008

**Date**	**Ellipse size of the epidemics**				**Moving direction of the center (Y axis)**	
	**Mild EV**		**Severe EV**		**Mild EV**	**Severe EV**
	**XStdDist (m)**	**YStdDist (m)**	**XStdDist (m)**	**YStdDist (m)**		
Apr-08	117475.15	273726.81	60763.89	232460.24	-	-
May-08	154384.39	264808.86	70947.83	239600.52	7881.29	20605.32
Jun-08	178337.48	271730.16	73179.24	247525.28	9265.09	29547.7
Jul-08	149327.52	270119.86	88125.03	204859.63	1845.9	-45949.13

## Discussion

Epidemics of enterovirus have continued playing a major public health threat in the Asia-Pacific region [22]. During the past decade, epidemics have also occurred in European countries, including Denmark [23], the United Kingdom [24], Hungary [25], France [26] and the Netherlands [27]. The integrated information from different enterovirus surveillance systems (rather than from a single source) plus spatio-temporal analyses of epidemiological data in Taiwan might provide valuable experience for other countries. Enhanced surveillance and non-pharmaceutical public health policy such as school closure have been the major strategies implemented for preventing enterovirus epidemics, because effective vaccines and antiviral drugs have not been available. In this study, we have shown that integrated surveillance, including sentinel physician-based clinical surveillance, virological surveillance, and notifiable infectious disease surveillance, reflected not only the whole spectrum of EV cases from mild to severe but also the types of virus activity at the community level. With the spatial scan and spatio-temporal scan statistics, we found that central Taiwan and Penghu County, which is an island located 44 km offshore, were the locations of major clusters of severe EV cases. In the temporal pattern, the severe EV cases occurred either one week earlier than the mild EV cases or surged simultaneously, during the same week. However, taking the autoregressive effects and EV isolation rates together, severe EV cases could not significantly explain the temporal trend of mild EV cases. In contrast, mild EV cases might have better prediction capability even after controlling for the AR effects.

In this study, the peak of the mild and the severe EV cases occurred almost the same week. In the years of 2000 and 2001, the increases of the mild EV cases occurred one to two weeks earlier than the increases of the severe EV cases. After 2002, the pattern changed to consistently high in the same week, or severe cases rising even much earlier. Several possible reasons might explain this phenomenon. First, the outbreak of SARS in 2003 made physicians in Taiwan more aware of unusual increases in case numbers of infectious diseases. Once severe EV cases were reported and announced through mass media, even at the early stage, it might have alerted the physicians to pay attention to additional suspected EV cases. Second, EV-71 was highly correlated with severe EV cases. This is consistent with the facts, that EV-71 was known to have high virulence, and pathogenicity in the central nervous system by inflammatory cytokines/chemokines [28,29], severe pulmonary edema and heart failure [30]. The early information on the positive isolation rate of EV-71 also can be helpful to predict the more likely occurrence of severe EV cases in the following one to two weeks.

With the space-time scan method, we found temporal clusters in June 2008, and spatial clusters in five cities and counties. One nearby island, Penghu, where the seashore is a famous summer tourist destination, had the highest local risk in 2000 and 2008. The possible reasons for this phenomenon might have been the smaller number of children amongst the population, lack of medical resources, and close transportation to another epidemic center, China.

The data in this study have three major limitations. Because of the de-identification between different released databases, direct linkage between the severe EV cases and the results of EV isolation was not feasible. Hence, it was hard to elucidate the correlation between local epidemics and the specific types of enterovirus circulated. Age-related data for mild EV incidence were not available in the sentinel surveillance database. Therefore, it was hard to differentiate among incidence in preschool children or school-aged children. Integration among different surveillance systems would be beneficial for disease surveillance. Public health surveillance and clinical surveillance could help detect aberrations at the early stage. Laboratory surveillance could help determine the severity of epidemics. In the future, utilizing community surveillance, hospital-based syndromic surveillance and National health insurance data, which cover 99% of the population in Taiwan, for monitoring age-specific enterovirus cases will offer the best chance to detect enterovirus activity with better sensitivity and timeliness at the local community level.

## Conclusions

Local public health professionals can monitor the temporal and spatial trends plus spatio-temporal clusters and isolation rate of EV-71 in mild and severe EV cases in the community when virus transmission is high to provide early warning signals and to prevent subsequent severe epidemics. The increase of mild EV cases might be a possible predictor for the occurrence of severe EV cases with a time series model. In addition, comprehensive surveillance of school children might detect earlier signals and allow social distance intervention to minimize the size of spatial clustering.

## Competing interests

The authors declare that we do not have any competing interests related to this study.

## Authors’ contributions

TCC did all spatio-temporal data analysis and writing for the whole manuscript. JSH participated in spatio-temporal statistics. RHC did most data analyses from surveillance by types of enteroviruses. CCK guided epidemiological study between mild and severe enterovirus cases and also the revision of this manuscript. PHC participated in GIS analysis and the revision of this manuscript. All authors read and approved the final manuscript.

## Pre-publication history

The pre-publication history for this paper can be accessed here:

http://www.biomedcentral.com/1471-2458/14/11/prepub
